# Mechanism of Taxanes in the Treatment of Lung Cancer Based on Network Pharmacology and Molecular Docking

**DOI:** 10.3390/cimb45080414

**Published:** 2023-08-07

**Authors:** Yajing Zhang, Zirui Zhao, Wenlong Li, Yuanhu Tang, Shujie Wang

**Affiliations:** College of Biological and Agricultural Engineering, Jilin University, Changchun 130022, China; zhangyj2304@163.com (Y.Z.); zzirui94@163.com (Z.Z.); liwl21@mails.jlu.edu.cn (W.L.); tang980501@163.com (Y.T.)

**Keywords:** taxanes, lung cancer, network pharmacology, molecular docking, target, pathway

## Abstract

Taxanes are natural compounds for the treatment of lung cancer, but the molecular mechanism behind the effects is unclear. In the present study, through network pharmacology and molecular docking, the mechanism of the target and pathway of taxanes in the treatment of lung cancer was studied. The taxanes targets were determined by PubChem database, and an effective compounds-targets network was constructed. The GeneCards database was used to determine the disease targets of lung cancer, and the intersection of compound targets and disease targets was obtained. The Protein–Protein Interaction (PPI) network of the intersection targets was analyzed, and the PPI network was constructed by Cytoscape 3.6.0 software. The hub targets were screened according to the degree value, and the binding activity between taxanes and hub targets was verified by molecular docking. The results showed that eight taxane-active compounds and 444 corresponding targets were screened out, and 131 intersection targets were obtained after mapping with lung cancer disease targets. The hub targets obtained by PPI analysis were TP53, EGFR, and AKT1. Gene Ontology (GO) biological function enrichment analysis obtained 1795 biological process (BP) terms, 101 cellular component (CC) terms, and 164 molecular function (MF) terms. There were 179 signaling pathways obtained by Kyoto Encyclopedia of Genes and Genomes (KEGG) pathway enrichment analysis. Twenty signaling pathways were screened out, mainly pathways in cancer, proteoglycans in cancer pathway, microRNAs in cancer pathway, and so on. Molecular docking shows that the binding energies of eight taxanes with TP53, EGFR, and AKT1 targets were less than −8.8 kcal/mol, taxanes acts on TP53, EGFR, and AKT1 targets through pathways in cancer, proteoglycans in cancer pathway and microRNAs in cancer pathway, and plays a role in treating lung cancer in biological functions such as protein binding, enzyme binding, and identical protein binding.

## 1. Introduction

Lung cancer, as one of the most commonly diagnosed malignant tumors and the leading causes of cancer death in the world, has a high incidence [1,2,3]. Because the early clinical symptoms of lung cancer are hidden, the best treatment opportunity has been lost when it is discovered, which makes the mortality of lung cancer high and difficult to cure, which has seriously endangered human life and brought a burden to public health all over the world [4,5].

In China, due to the influence of regional differences, lung cancer is the malignant tumor with the highest mortality rate, although its impact on different regions is different [6,7]. At the same time, lung cancer is also the most common cancer that affects the quality of life of both sexes in China [8]. Although diagnostic techniques and treatment methods are developing rapidly, the incidence and mortality of lung cancer are rising [9].

Lung cancer can be divided into small cell lung cancer (SCLC) and non-small cell lung cancer (NSCLC) according to its pathological type, cell morphology, and histological characteristics. Taxanes have a very significant therapeutic effect on lung cancer [10,11]. Taxanes are a series of derivatives which are isolated from *Taxus* plants. Taxanes are a kind of diterpenoids with pentamethyl pentadecene skeleton, taxanes mainly include paclitaxel, docetaxel, and derivatives with taxanes skeleton structure, which have high-efficiency and broad-spectrum anti-tumor activity, and their development has made great contributions to drug discovery and clinical application [12]. Some taxanes, such as 10-deacetylbaccatin III (10-DAB III), can be used as raw materials for semi-synthetic paclitaxel, while others, such as docetaxel, 7-xylosyl-10-deacetyltaxol (7-Xyl-10-DAT), and so on, have various pharmacological activities [13,14]. Paclitaxel is used to treat SCLC, for example, Annic et al.’s research shows that epirubicin combined with paclitaxel can be used to treat SCLC after platinum chemotherapy failure [15]. Wang et al. shows that nanoparticle albumin combined with paclitaxel is safe and effective in the third-line treatment of recurrent SCLC [16], and Eiff et al.’s research shows that paclitaxel has clinically relevant activity in heavily pretreated SCLC [17]. In addition, studies have shown that taxanes are used to treat NSCLC. Francesco et al. shows that two-drug combinations consisting of either cisplatin or carboplatin plus a third-generation agent (docetaxel, paclitaxel, gemcitabine, or vinorelbine) remain the primary treatment option for advanced NSCLC patients [18]. Liu et al., provided comparisons to integrate the efficacy of immunotherapy and taxane chemotherapy as second- or later-line treatments in advanced NSCLC, and the combination therapy showed the most favorable progression-free survival (PFS) [19]. Fanucchi et al. shows that paclitaxel and docetaxel, drugs that bind tightly to β-tubulin and disrupt microtubule dynamics, are widely used in the treatment of NSCLC [20]. As the first promoter of tubulin polymerization, taxanes play an important role in the war against cancer and the exploration of new microtubule-binding drugs [21]. When cancer cells divide, they can combine with cell microtubule proteins, promote the stability and polymerization of microtubules in cells, prevent microtubule depolymerization, and block cell division [22].

As one of the most widely used and researched natural anticancer compounds, taxanes play an important role in clinic, practice, and drug development [23,24]. Previous studies have shown that taxanes have an obvious inhibitory effect on lung cancer tumor cells [25,26], and the anti-tumor effect of taxanes is the result of multiple mechanisms. Different types of cancer cell lines and different experimental conditions have different degrees of influence on the anti-cancer mechanism of taxanes [27,28,29].

Lung cancer has a high mortality rate worldwide, which seriously endangers human life, health, and safety. Taxanes are one of the most common compounds for treating lung cancer, so far there is no systematic theory that can fully explain the anti-lung cancer mechanism of taxanes. Network pharmacology is a new subject based on the theory of systems biology [30,31]. Using statistics, network visualization and other means, it can better build a multi-level network at the molecular level, explore the correlation between compounds and diseases [32], explain the research methods of compound action mechanism from the overall level, and provide new perspectives and explanations for compounds action mechanism [33,34,35]. Molecular docking is a method of drug design through the characteristics of receptors and the interaction between receptors and drug molecules [36]. It is a theoretical simulation method that mainly studies the interaction between molecules (such as ligands and receptors) and predicts their binding patterns and affinities [37]. Molecular docking technology can greatly improve the work efficiency and reduce the research cost. It has become an important tool to predict the binding affinity and analyze the interaction mode in computer-aided drug design, and is of great significance to predict the molecular mechanism of pharmacological activities of small molecular compounds [38].

In the present study, taxanes and lung cancer were taken as the research objects, and the effective compounds, hub targets, core pathways, and core functions of taxanes in the treatment of lung cancer were screened and analyzed by using network pharmacology technology. Through the Gene Ontology (GO) enrichment analysis and Kyoto Encyclopedia of Genes and Genomes (KEGG), the pathway enrichment analysis of intersection targets was carried out, and the hub targets were verified by molecular docking technology, aiming to preliminarily clarify the molecular mechanism of taxanes in the treatment of lung cancer from the perspective of network pharmacology.

## 2. Materials and Methods

### 2.1. Screening of Effective Active Compounds of Taxanes and Construction of Compounds-Targets

The literatures related to taxanes were searched through China National Knowledge Infrastructure (https://www.cnki.net/, accessed on 6 May 2022), and the collected taxanes mainly contained eight compounds (10-deacetylbaccatin III (10-DAB III), baccatin III, 7-xylosyl-10-deacetyltaxol (7-Xyl-10-DAT), 10-deacetyltaxol (10-DAT), docetaxel, cephalomannine (CEPH), paclitaxel, and 7-epitaxol). Eight typical taxane targets were obtained by using PubChem database (https://pubchem.ncbi.nlm.nih.gov/, accessed on 14 May 2022), and the taxane targets were matched with gene names by using Uniprot database of protein sequence and functional information resources (http://www.uniprot.org/, accessed on 18 May 2022), and repeated values were deleted to obtain taxane targets. Upload the data to Cytoscape 3.6.0 software to draw the effective compounds-targets network diagram.

### 2.2. Screening of Lung Cancer Disease Targets

We used GeneCards disease database (https://www.genecards.org/, accessed on 25 May 2022), searched with “lung cancer” as the key word, and screened with “relevance score” ≥ 25 to obtain disease targets.

### 2.3. Acquisition of Intersection Targets and Construction of PPI Network

We calculated and drew a custom Venn diagram on the website of Bioinformatics Evolutionary Genomics (http://bioinformatics.psb.ugent.be/webtools/Venn/, accessed on 29 May 2022) of the intersection targets of eight taxanes and lung cancer, and the intersection targets of eight taxanes and lung cancer was obtained. And the intersection targets were imported into the STRING database (https://string-db.org/, accessed on 6 June 2022) for Protein–Protein Interaction (PPI) network analysis, and the species was defined as “Homo Sapiens”, and the “minimum required interaction score” was set to 0.4 to construct a PPI network. The PPI network map was downloaded in “TSV” format and uploaded to the Cytoscape 3.6.0 software to make a PPI network visualization.

### 2.4. Analysis of GO and KEGG Pathway Enrichment

The GO function and KEGG pathway of the intersection targets were analyzed by Metascape database (http://metascape.org/gp/index.html#/main/step1, accessed on 15 February 2023), and the selected species was “Homo sapiens”. GO function analysis mainly includes biological process (BP), cellular component (CC), and molecular function (MF) terms. The threshold of statistical significance was set to *p* < 0.001, and the data results were visualized by using the free bioinformatics database (http://bioinformatics.com.cn/, accessed on 26 February 2023). The drawing of the core pathway picture depends on KEGG Mapper–Color website (https://www.genome.jp/kegg/mapper/color.html, accessed on 2 March 2023).

### 2.5. Molecular Docking

In order to further analyze the research results of network pharmacology at the level of molecular relationship, with the help of molecular docking technology [39], the top three hub targets in PPI network relationship were selected as receptors, and eight taxanes were used as ligands for molecular docking. Three-dimensional structural information of eight taxanes was obtained through PubChem database (https://pubchem.ncbi.nlm.nih.gov/, accessed on 3 March 2023), and it was saved as “SDF” format. Based on PPI network analysis results (the top three hub targets with higher degree values), the crystal structures of TP53 (6MXY, resolution: 1.624 Å), EGFR (3POZ, resolution: 1.5 Å), and AKT1 (3OS5, resolution: 1.69 Å) were chosen as the receptor proteins from the RCSB protein data bank (https://www.rcsb.org/, accessed on 4 March 2023), and then the “SDF” format of eight taxanes is converted into “PDB” format by using OpenBabel 2.4.1 software. Autodock 4.2 software was introduced, and the small molecule and target protein were pretreated by OpenBabel 2.4.1 software, respectively, and the treated compounds were molecularly docked with the target protein by PyMOL 2.4 software. The binding energy of the compounds and targets were then determined. The binding sites were visualized on Protein–Ligand Interaction Profiler (PLIP) website (https://projects.biotec.tu-dresden.de/plip-web/plip/index, accessed on 14 March 2023). And determination of RMSD values of the compounds and targets by “align” function of PyMOL 2.4 software.

## 3. Results

### 3.1. Construction of Compounds-Targets Network

**Figure 1:**
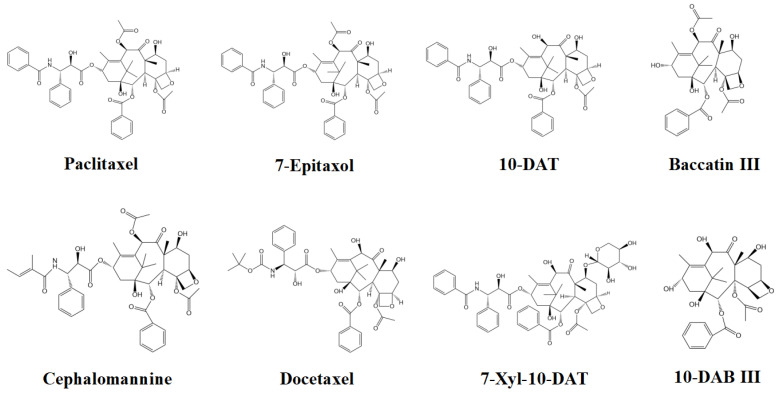
Chemical structures of the eight taxanes.

**Figure 2:**
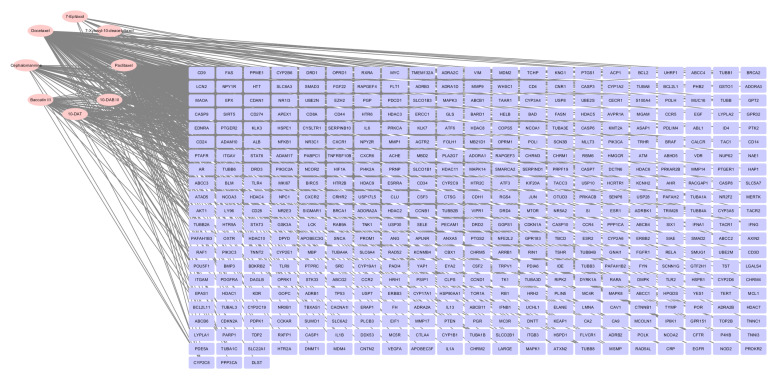
Network diagram of eight taxanes related targets. The pink nodes represent eight taxanes, and purple nodes represent the targets corresponding to eight taxanes.

Figure 1 shows the chemical structural formulas of eight taxanes. Eight taxane targets were obtained through PubChem database (https://pubchem.ncbi.nlm.nih.gov/, accessed on 14 May 2022), and the targets of effective compounds were matched with human gene names by Uniprot database (http://www.uniprot.org/, accessed on 18 May 2022). After deleting duplicate values, a total of 444 taxanes-related targets were obtained, including 444 targets for paclitaxel, 40 targets for 10-deacetylbaccatin III (10-DAB III), 40 targets for 10-deacetyltaxol (10-DAT), 48 targets for baccatin III, 48 targets for cephalomannine (CEPH), 147 targets for docetaxel, 10 targets for 7-epitaxol, and 5 targets for 7-xylosyl-10-deacetyltaxol (7-Xyl-10-DAT). Visualize with the “Analyza Network” tool in cytoscape 3.6.0 software (Figure 2). From Figure 2, the pink elliptic nodes on the left represent eight taxanes, and the purple rectangular nodes on the right represents 444 targets corresponding to eight taxanes.

### 3.2. Screening of Lung Cancer Disease Targets and Construction of PPI Network of Intersection Targets

Screening of lung cancer disease targets Using GeneCards disease database (https://www.genecards.org/, accessed on 25 May 2022), we searched with “lung cancer” as the key word and screened with “Relevance score” ≥ 25” to obtain disease targets, and a total of 738 lung cancer-related targets were screened.

**Figure 3:**
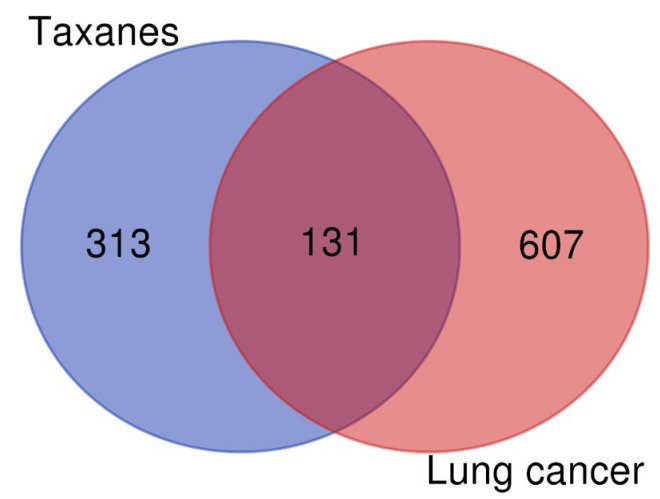
Venn diagram of eight taxane targets and lung cancer targets.

As shown in Figure 3, the lung-cancer-related targets and the taxanes target were mapped to each other using the Bioinformatics Evolutionary Genomics (http://bioinformatics.psb.ugent.be/webtools/Venn/, accessed on 29 May 2022), blue represents eight taxanes with 444 targets, red represents lung cancer with 738 targets, and the middle overlapping part was the intersection target of eight taxanes and lung cancer with 131 targets.

**Figure 4:**
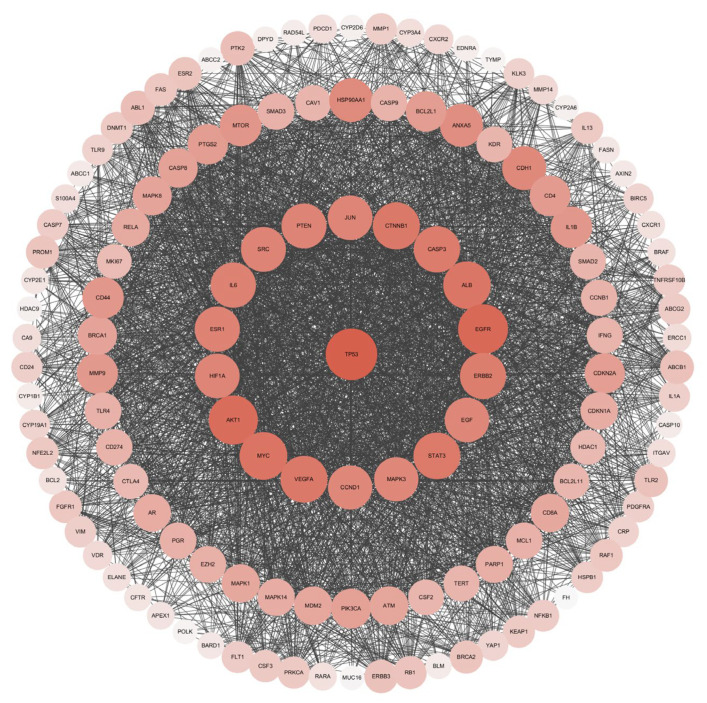
PPI network visualization of intersection target of eight taxanes and lung cancer.

Figure 4 represents the PPI network visualization of 131 intersection target of eight taxanes and lung cancer. The results showed that the network contains 131 nodes and 3284 edges, and the average node degree is 50.14. Among them, 131 nodes represent 131 targets, and 3284 edges represent 3284 related information among targets, with an average node degree of 50.14, which means that each node in the active ingredient target-disease target network is connected with an average of 50.14 edges. The size and color of the node are positively correlated with the degree value. The greater the degree value, the larger the node and the darker the color. After analyzing the results (Table 1), targets TP53, EGFR, AKT1, MYC, ALB, CTNNB1, VEGFA, STAT3, CASP3, ERBB2, IL6, ESR1, PTEN, SRC, JUN, HIF1A, EGF, CCND1, and MAPK3 showed a high degree value (≥90). The top three targets with the highest correlation are TP53, EGFR, and AKT1, and the degree values were 121, 114, and 111, respectively. TP53 is a P53 protein encoded by TP53 (Tumor protein p53) gene, which mainly regulates cell division and proliferation, and is also a tumor suppressor gene. Epidermal growth factor receptor (EGFR) is a tyrosine kinase receptor for epithelial growth factor (EGF) cell proliferation and signal transduction, belonging to the ErbB receptor family, which is related to tumor cell proliferation, angiogenesis, tumor invasion, metastasis, and apoptosis inhibition. RAC-alpha serine/threonine-protein kinase (AKT1) gene encodes serine/threonine protein kinase, which can be activated by extracellular signal through phosphatidylinositol 3 kinase (PI3K)-dependent mechanism.

### 3.3. GO Biological Function Enrichment

**Figure 5:**
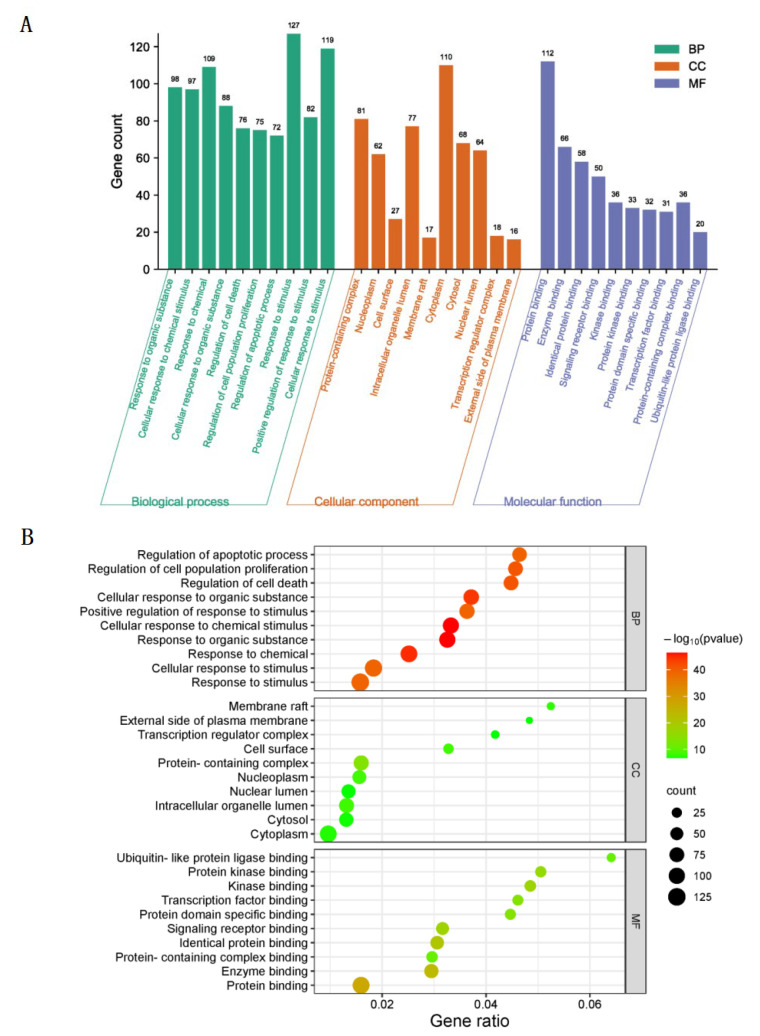
GO enrichment analysis of intersection targets. (**A**) Histogram of intersection targets, (**B**) bubble graph of intersection targets (*p* < 0.001).

GO biological functions of 131 intersection target of taxanes and lung cancer genes were analyzed by Metascape database (http://metascape.org/gp/index.html#/main/step1, accessed on 15 February 2023), and 1795 biological process (BP) terms, 101 cellular component (CC) terms, and 164 molecular function (MF) terms were obtained. *p* < 0.001 was set as the screening standard, and the top 10 biological process terms with the highest correlation were selected, respectively. As shown in Figure 5A, the ordinate of the histogram represents the number of genes enriched in each pathway, and the abscissa represents the name of GO. As shown in Figure 5B, the ordinate of the bubble graph represents the name of the channel, the abscissa represents the proportion of genes enriched in each channel, the size of the circle represents the significance of enrichment, and the darker the color, the more significant the genes in the network are enriched in this channel. The results showed that among the intersection target of taxanes and lung cancer, BP terms mainly focuses on response to stimulus, cellular response to stimulus, response to chemical, response to organic substance, cellular response to chemical stimulus, cellular response to organic substance, positive regulation of response to stimulus, regulation of cell death, regulation of cell population proliferation, and regulation of apoptotic process. CC terms mainly focus on cytoplasm, protein-containing complex, intracellular organelle lumen, cytosol, nuclear lumen, nucleoplasm, cell surface, transcription regulator complex, membrane raft and external side of plasma membrane. MF terms mainly focus on protein binding, enzyme binding, identical protein binding, signaling receptor binding, kinase binding, protein-containing complex binding, protein kinase binding, protein domain specific binding, transcription factor binding and ubiquitin-like protein ligase binding.

### 3.4. KEGG Pathway Enrichment

**Figure 6:**
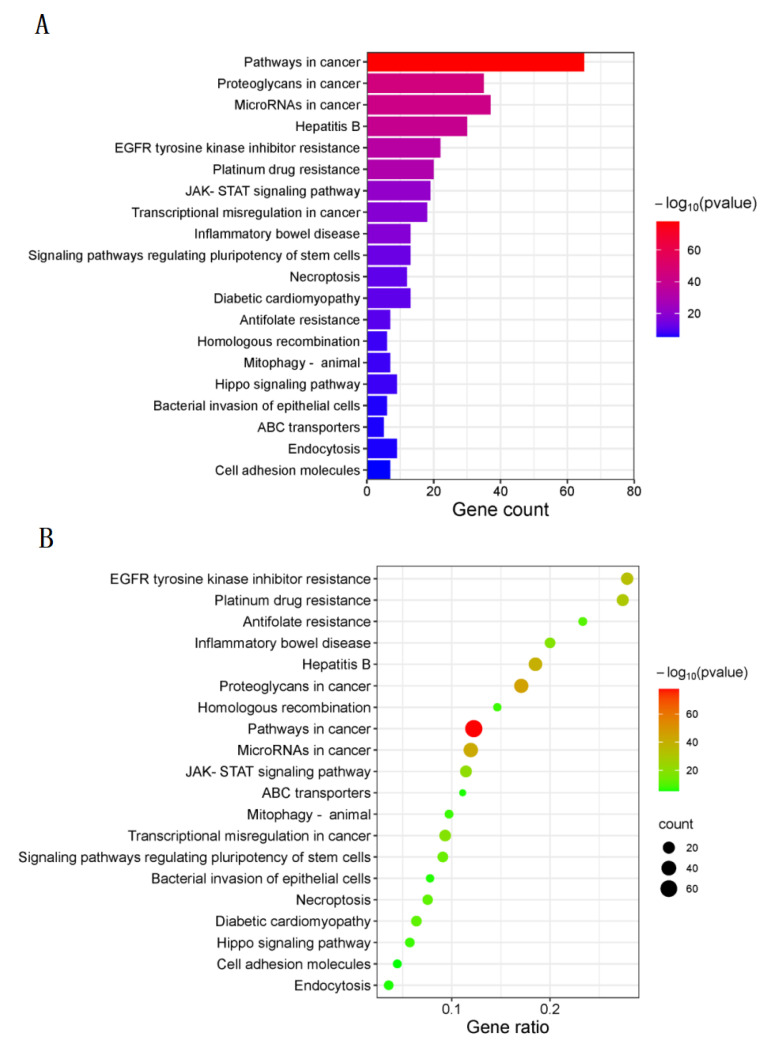
KEGG pathway enrichment of intersection targets. (**A**) Histogram of intersection targets, (**B**) bubble graph of intersection targets (*p* < 0.001).

A total of 131 intersection targets of taxanes and lung cancer genes were uploaded to Metascape database (http://metascape.org/gp/index.html#/main/step1, accessed on 15 February 2023), and KEGG pathway enrichment analysis was carried out. There were 179 signaling pathways obtained by KEGG pathway enrichment analysis and the top 20 signaling pathways with the highest correlation were screened out (*p* < 0.001). As shown in Figure 6A, the ordinate of the histogram represents the name of the pathway, the abscissa represents the number of genes enriched in each pathway, and the color represents the significance of enrichment. The darker the color, the longer the band, the more genes in the network are enriched in this pathway. As shown in Figure 6B, the ordinate of the bubble graph represents the name of the channel, the abscissa represents the proportion of genes enriched in each channel, the size of the circle represents the significance of enrichment, the larger the circle and the darker the color, the more significant the genes in the network are enriched in this channel. The results showed that the first three pathways with the highest enrichment significance were pathways in cancer, proteoglycans in cancer and microRNAs in cancer. Thus, taxanes may intervene lung cancer by regulating multiple signaling pathways.

**Figure 7:**
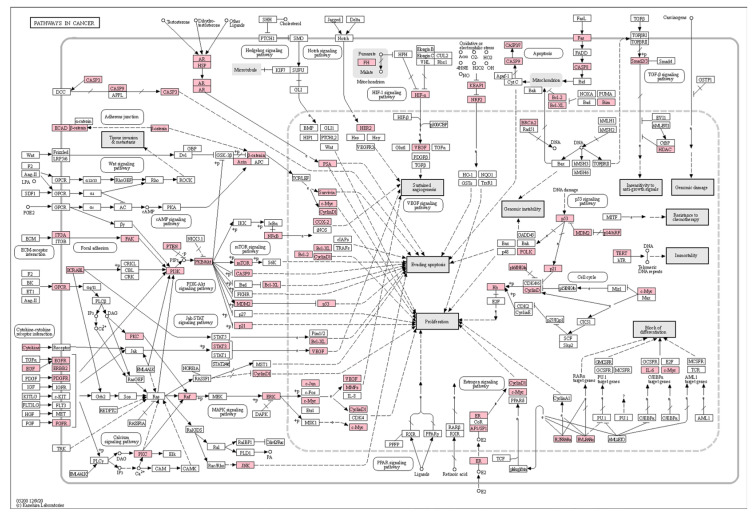
Pathways in cancer core pathway diagram and main targets. The intersection targets were marked in pink.

The analysis of KEGG pathway-enriched genes shows that the first three targets mentioned above are TP53, EGFR, and AKT1 according to the degree value (Table 1). They are all enriched in Pathways in cancer signaling pathway, so it can be seen that Pathways in cancer pathway is one of the important pathways to explore the treatment of lung cancer with taxanes. The core pathway diagram is shown in Figure 7, and the target of action in the pathway is marked in white, and the intersection target of taxanes and lung cancer is marked in pink.

### 3.5. Molecular Docking

Topological analysis results of the intersection targets showed that targets of TP53, EGFR, and AKT1 had a higher “degree value” and “closeness centrality”, as shown in Table 1. Thus, targets TP53, EGFR, and AKT1 were chosen for molecular docking to further verify the correctness of the above analysis. Eight taxanes were used as ligands, and the top three intersection targets in PPI network relationship were selected as receptors for molecular docking.

**Figure 8:**
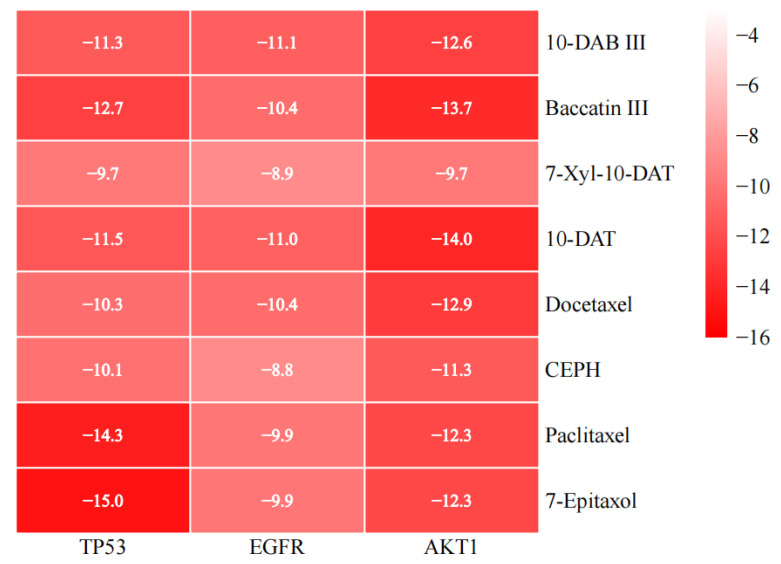
Heat map of molecular docking binding energy of taxanes with corresponding hub targets, unit (kcal/mol).

Eight taxanes were molecularly docked with three hub targets TP53 (6MXY), EGFR (3POZ), and AKT1 (3OS5), respectively. Twenty-four groups of ligand-receptor docking results were obtained (Table 2). Targets with good binding activity were screened according to the binding energy, and the lower the binding energy, the greater the binding force between the ligand and the receptor; the binding energy was lower than −5 kcal/mol, which indicates that the ligand and the receptor can bind well [40]. Among them, the binding energies of 10-DAB III with TP53, EGFR, and AKT1 were −11.3, −11.1, and −12.6 kcal/mol, respectively; the binding energies of baccatin III with TP53, EGFR, and AKT1 were −12.7, −10.4, and −13.7 kcal/mol, respectively; and the binding energies of 7-Xyl-10-DAT with TP53, EGFR, and AKT1 were −9.7, −8.9, and −9.7 kcal/mol, respectively. The binding energies of 10-DAT with TP53, EGFR, and AKT1 were −11.5, −11.0, and −14.0 kcal/mol, respectively; the binding energies of docetaxel with TP53, EGFR, and AKT1 were −10.3, −10.4, and −12.9 kcal/mol, respectively; and the binding energies of CEPH with TP53, EGFR, and AKT1 were −10.1 and −8.8, −11.3 kcal/mol, respectively. The binding energies of paclitaxel with TP53, EGFR, and AKT1 were −14.3, −9.9, and −12.3 kcal/mol, respectively, and the binding energies of 7-epitaxol with TP53, EGFR, and AKT1 were −15.0, −9.9, and −12.3 kcal/mol, respectively. The docking results were displayed in the form of heat map (Figure 8), and the binding energy is negatively correlated with the affinity between ligands and receptors.

According to the binding energies, the above eight compounds can be well-combined with the three targets. 10-DAB III has the strongest binding with AKT1 target, followed by TP53 target and the weakest binding with EGFR target. Baccatin III has the strongest binding with AKT1 target, followed by TP53 target and the weakest binding with EGFR target. 7-Xyl-10-DAT has the strongest binding with TP53 and AKT1 targets, and the weakest binding with EGFR targets. 10-DAT has the strongest binding with AKT1 target, followed by TP53 target and the weakest binding with EGFR target. Docetaxel has the strongest binding with AKT1 target, followed by EGFR target and the weakest binding with TP53 target. CEPH has the strongest binding with AKT1 target, followed by TP53 target and the weakest binding with EGFR target. Paclitaxel has the strongest binding with TP53 target, followed by AKT1 target and the weakest binding with EGFR target. 7-epitaxol has the strongest binding with TP53 target, followed by AKT1 target and the weakest binding with EGFR target.

**Figure 9:**
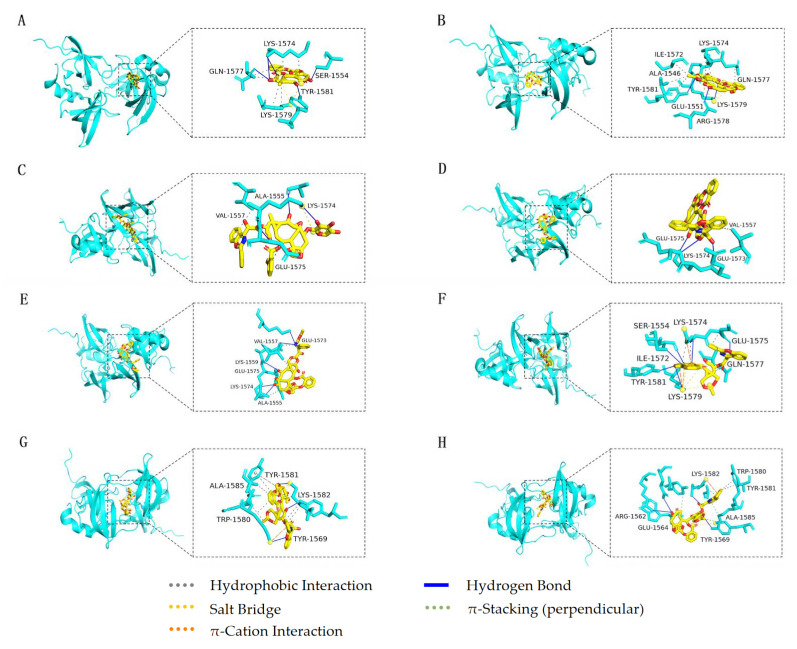
Molecular docking and binding sites of eight taxanes and TP53 target (**A**) 10-DAB III (yellow) and TP53 target (cyan), (**B**) baccatin III (yellow) and TP53 target (cyan), (**C**) 7-Xyl-10-DAT (yellow) and TP53 target (cyan), (**D**) 10-DAT (yellow) and TP53 target (cyan), (**E**) docetaxel (yellow) and TP53 target (cyan), (**F**) CEPH (yellow) and TP53 target (cyan), (**G**) paclitaxel (yellow) and TP53 target (cyan), and (**H**) 7-epitaxol (yellow) and TP53 target (cyan).

As shown in Figure 9A, 10-DAB III forms two hydrogen bonds with amino acid residues SER1554 and GLN1577 of TP53, four hydrogen bonds with residues LYS1574 and TYR1581, six hydrophobic interactions with residues LYS1574, GLN1577, and LYS1579, and 10-DAB III forms two salt bridges with residues LYS1579. After docking 10-DAB III with TP53 target, the root mean square deviation (RMSD) value was 0.366 Å. As shown in Figure 9B, Baccatin III forms four hydrogen bonds with amino acid residues GLU1551, ARG1578, LYS1579, and TYR1581 of TP53, five hydrophobic interactions with residues ALA1546, ILE1572, GLN1577, LYS1579, and TYR1581, and two hydrophobic interactions with residue LYS1574. In addition, baccatin III forms three salt bridges with residue LYS1579. After docking 10-DAB III with TP53 target, the RMSD value was 0.371 Å. As shown in Figure 9C, 7-Xyl-10-DAT forms three hydrogen bonds with amino acid residues ALA1555, LYS1574, and GLU1575 of TP53, and two hydrophobic interactions with residues VAL1557 and GLU1575. In addition, 7-Xyl-10-DAT forms a salt bridge with residue LYS1574. After docking 7-Xyl-10-DAT with TP53 target, the RMSD value was 0.390 Å. As shown in Figure 9D, 10-DAT forms three hydrogen bonds with amino acid residues GLU1573, LYS1574, and GLU1575 of TP53, and 10-DAT forms a hydrophobic interaction with residue VAL1557. After docking 10-DAT with TP53 target, the RMSD value was 1.508 Å. As shown in Figure 9E, docetaxel forms four hydrogen bonds with amino acid residues ALA1555, LYS1559, LYS1574, and GLU1575 of TP53, and two hydrogen bonds with GLU1573. In addition, docetaxel forms a hydrophobic interaction with residue ALA1555 and two hydrophobic interactions with residue VAL1557. After docking docetaxel with TP53 target, the RMSD value was 0.662 Å. As shown in Figure 9F, CEPH forms six hydrogen bonds with amino acid residues SER1554, ILE1572, LYS1574, GLU1575, LYS1579, and TYR1581 of TP53, three hydrophobic interactions with residues GLU1575, GLN1577, and LYS1579, and two hydrophobic interactions with LYS1574. In addition, CEPH forms two π-cation interactions with residue LYS1579, one π-cation interaction with residue LYS1574, and three salt bridges with residue LYS1579. After docking CEPH with TP53 target, the RMSD value was 0.460 Å. As shown in Figure 9G, paclitaxel forms four hydrogen bonds with amino acid residues TYR1569 and LYS1582 of TP53, three hydrophobic interactions with residues TRP1580, TYR1581, and ALA1585, and five hydrophobic interactions with LYS1582. In addition, paclitaxel forms a π-stacking (perpendicular) with residue TYR1581, a π-cation interaction with residue LYS1582, and two salt bridges with residue LYS1582. After docking paclitaxel with TP53 target, the RMSD value was 0.443 Å. As shown in Figure 9H, 7-epitaxol forms four hydrogen bonds with amino acid residues ARG1562, GLU1564, TYR1569, and LYS1582 of TP53, six hydrophobic interactions with residues TYR1569, LYS1582, and ALA1585, and two hydrophobic interactions with TRP1580 and TYR1581. In addition, 7-epitaxol forms a π-cation interaction and a salt bridge with residue LYS1582. After docking 7-epitaxol with TP53 target, the RMSD value was 0.636 Å.

**Figure 10:**
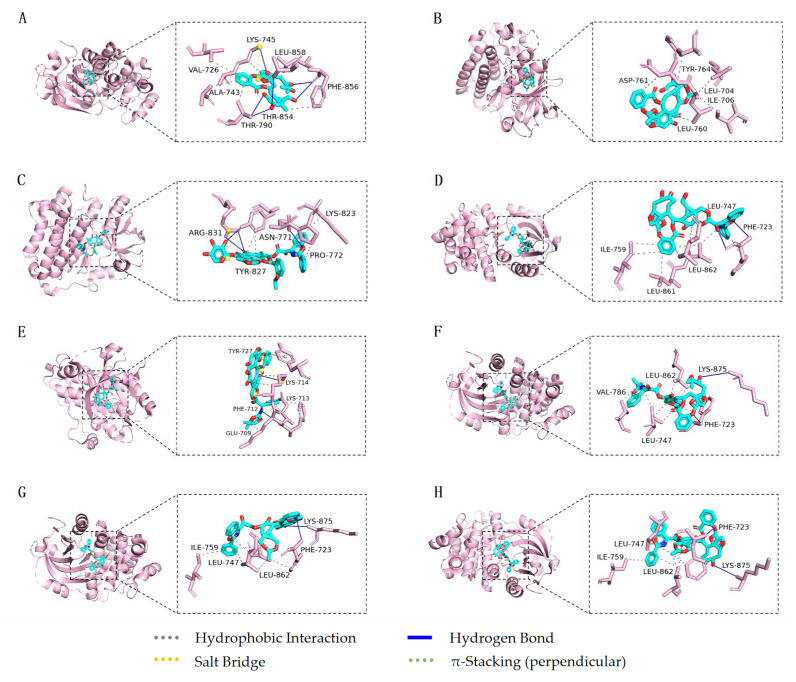
Molecular docking and binding sites of eight taxanes and EGFR target (**A**) 10-DAB III (cyan) and EGFR target (lightpink), (**B**) baccatin III (cyan) and EGFR target (lightpink), (**C**) 7-Xyl-10-DAT (cyan) and EGFR target (lightpink), (**D**) 10-DAT (cyan) and EGFR target (lightpink), (**E**) docetaxel (cyan) and EGFR target (lightpink), (**F**) CEPH (cyan) and EGFR target (lightpink), (**G**) paclitaxel (cyan) and EGFR target (lightpink), and (**H**) 7-epitaxol (cyan) and EGFR target (lightpink).

As shown in Figure 10A, 10-DAB III forms two hydrogen bonds with amino acid residue THR790 of EGFR, two hydrogen bonds with residue PHE856, two hydrogen bonds with LYS745 and THR854, two hydrophobic interactions with residue LYS745, and five hydrophobic interactions with residues VAL726, ALA743, THR790, PHE856, and LEU858. After docking 10-DAB III with EGFR target, the RMSD value was 0.204 Å. As shown in Figure 10B, baccatin III forms two hydrophobic interactions with the amino acid residue LEU760 of EGFR, and four hydrophobic interactions with LEU704, ILE706, ASP761, and TYR764. After docking baccatin III with EGFR target, the RMSD value was 0.324 Å. As shown in Figure 10C, 7-Xyl-10-DAT forms two hydrogen bonds with amino acid residue ARG831 of EGFR, two hydrogen bonds with ASN771 and TYR827, two hydrophobic interactions with TYR827, and two hydrophobic interactions with PRO772 and LYS823. In addition, 7-Xyl-10-DAT forms a salt bridge with residue ARG831. After docking 7-Xyl-10-DAT with EGFR target, the RMSD value was 0.845 Å. As shown in Figure 10D, 10-DAT forms two hydrogen bonds with amino acid residue PHE723 of EGFR, two hydrophobic interactions with residue LEU747, two hydrophobic interactions with residue ILE759, two hydrophobic interactions with residue LEU862, and two hydrophobic interactions with residues PHE723 and LEU861. After docking 10-DAT with EGFR target, the RMSD value was 0.680 Å. As shown in Figure 10E, docetaxel forms two hydrogen bonds with amino acid residues PHE712 and LYS714 of EGFR, two hydrophobic interactions with residue LYS714, and four hydrophobic interactions with residues GLU709, PHE712, LYS713, and TYR727. In addition, docetaxel forms π-stacking (perpendicular) with residue TYR727 and three salt bridges with residue LYS714. After docking docetaxel with EGFR target, the RMSD value was 0.857 Å. As shown in Figure 10F, CEPH forms two hydrogen bonds with amino acid residues PHE723 and LYS875 of EGFR, three hydrophobic interactions with residue LEU747, two hydrophobic interactions with residue LEU862 and two hydrophobic interactions with residue PHE723 and VAL786. After docking CEPH with EGFR target, the RMSD value was 0.408 Å. As shown in Figure 10G, paclitaxel forms two hydrogen bonds with amino acid residues PHE723 and LYS875 of EGFR, three hydrophobic interactions with residue LEU747, three hydrophobic interactions with residue LEU862 and two hydrophobic interactions with residue PHE723 and ILE759. After docking paclitaxel with EGFR target, the RMSD value was 0.509 Å. As shown in Figure 10H, 7-epitaxol forms two hydrogen bonds with amino acid residues PHE723 and LYS875 of EGFR, three hydrophobic interactions with residue LEU862, two hydrophobic interactions with residue LEU747 and two hydrophobic interactions with residue PHE723 and ILE759. After docking 7-epitaxol with EGFR target, the RMSD value was 0.272 Å.

**Figure 11:**
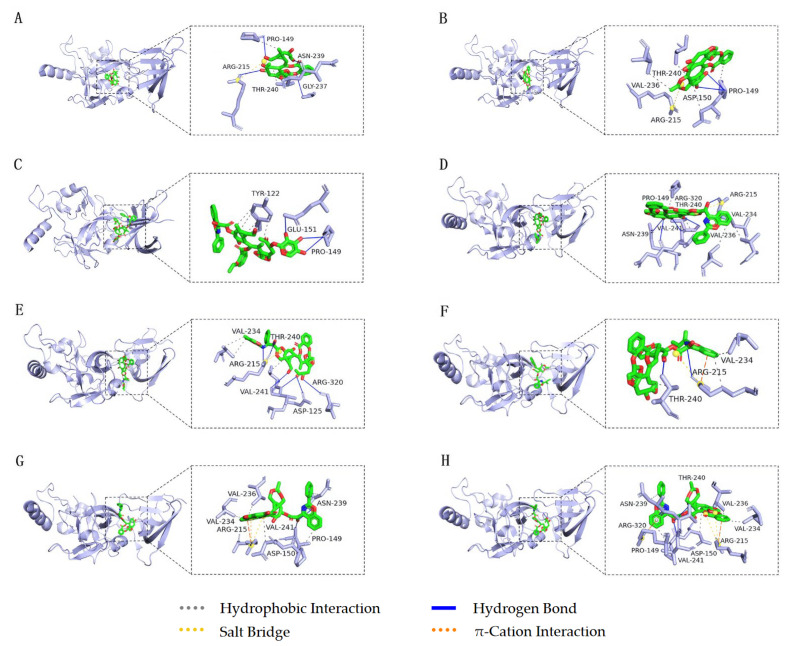
Molecular docking and binding sites of eight taxanes and AKT1 target (**A**) 10-DAB III (green) and AKT1 target (lightblue), (**B**) baccatin III (green) and AKT1 target (lightblue), (**C**) 7-Xyl-10-DAT (green) and AKT1 target (lightblue), (**D**) 10-DAT (green) and AKT1 target (lightblue), (**E**) docetaxel (green) and AKT1 target (lightblue), (**F**) CEPH (green) and AKT1 target (lightblue), (**G**) paclitaxel (green) and AKT1 target (lightblue), and (**H**) 7-epitaxol (green) and AKT1 target (lightblue).

As shown in Figure 11A, 10-DAB III forms four hydrogen bonds with amino acid residues PRO149, GLY237, ASN239, and ARG215 of AKT1, and two hydrophobic interactions with residues PRO149 and THR240. In addition, 10-DAB III forms a salt bridge with residue ARG215. After docking 10-DAB III with AKT1 target, the RMSD value was 0.363 Å. As shown in Figure 11B, baccatin III forms two hydrogen bonds with amino acid residue PRO149 of AKT1, four hydrophobic interactions with residues PRO149, ASP150, VAL236, and THR240, and a salt bridge with residue ARG215. After docking baccatin III with AKT1 target, the RMSD value was 0.093 Å. As shown in Figure 11C, 7-Xyl-10-DAT forms one hydrogen bond with amino acid residue GLU151 of AKT1, two hydrogen bonds with residue PRO149, and four hydrophobic interactions with residue TYR122. After docking 7-Xyl-10-DAT with AKT1 target, the RMSD value was 0.949 Å. As shown in Figure 11D, 10-DAT forms three hydrogen bonds with amino acid residues ARG215, ASN239, and ARG320 of AKT1, two hydrogen bonds with VAL241, four hydrophobic interactions with residues PRO149, VAL234, VAL236, and THR240, and a salt bridge with residue ARG215. After docking 10-DAT with AKT1 target, the RMSD value was 0.335 Å. As shown in Figure 11E, docetaxel forms two hydrogen bonds with amino acid residues ASP125 and ARG320 of AKT1, four hydrogen bonds with ARG215 and VAL241, two hydrophobic interactions with VAL234 and a hydrophobic interaction with THR240. In addition, docetaxel forms a salt bridge with residue ARG215. After docking docetaxel with AKT1 target, the RMSD value was 0.595 Å. As shown in Figure 11F, CEPH forms two hydrogen bonds with amino acid residues ARG215 and THR240 of AKT1, and three hydrophobic interactions with residues ARG215, VAL234, and THR240. In addition, CEPH forms a π-cation interaction and a salt bridge with residue ARG215. After docking CEPH with AKT1 target, the RMSD value was 0.482 Å. As shown in Figure 11G, paclitaxel forms two hydrogen bonds with amino acid residues ASN239 and VAL241 of AKT1, and five hydrophobic interactions with residues PRO149, ASP150, VAL234, VAL236, and ASN239. In addition, paclitaxel forms a π-cation interaction with residue ARG215 and two salt bridges with residue ARG215. After docking paclitaxel with AKT1 target, the RMSD value was 0.474 Å. As shown in Figure 11H, 7-epitaxol forms two hydrogen bonds with amino acid residues THR240 and VAL241 of AKT1, five hydrophobic interactions with residues PRO149, ASP150, VAL234, VAL236, and ASN239, and two salt bridges with residue ARG215. In addition, 7-epitaxol forms two π-cation interactions with residues ARG215 and ARG320. After docking 7-epitaxol with AKT1 target, the RMSD value was 0.477 Å.

## 4. Discussion

Lung cancer is the most frequent cancer type and the leading cause of tumor-associated deaths worldwide. The Tumor protein P53 (TP53) gene is a major player in cancer formation, and it is considered the most important tumor suppressor gene. The p53 protein acts as a transcription factor, and it is involved in DNA repair, senescence, cell-cycle control, autophagy, and apoptosis [41]. TP53 gene has anti-inflammatory activity and is often inactivated in lung cancer [42]. Studies have shown that the expression and function of P53 can be down-regulated during the invasion and metastasis of lung cancer [43]. Increasing the expression of P53 can enhance anti-inflammatory activity and play an important role in improving inflammation and tumor-related inflammation [44].

Epidermal growth factor receptor (EGFR) is a tyrosine kinase receptor for epithelial growth factor (EGF) cell proliferation and signal transduction, belonging to the ErbB receptor family, which is related to tumor cell proliferation, angiogenesis, tumor invasion, metastasis, and apoptosis inhibition [45]. EGFR is highly expressed in certain cancer types and is involved in regulating the biological characteristics of cancer progression, including proliferation, metastasis, and drug resistance. Various medicines targeting EGFR have been developed and approved for several cancer types, such as lung and colon cancer [46]. EGFR is related to the pathogenesis of NSCLC [47]. Studies have proved that EGFR has a unique potential epigenetic regulation through Long non-coding RNAs (lncRNAs) in genes [48]. Other studies have shown that the re-expression of EGFR in MTLn3-paxillinS178A rescued spontaneous metastasis of breast cancer patients from breast to lung [49].

RAC-alpha serine/threonine-protein kinase (AKT1) gene encodes serine/threonine protein kinase, which can be activated by extracellular signal through phosphatidylinositol 3 kinase (PI3K)-dependent mechanism, and participates in cellular processes, including apoptosis and glucose metabolism [50]. AKT1 plays a key role in cell growth and survival, and its activation in tumor is mediated by different mechanisms, including the increase in gene expression and protein phosphorylation after translation. AKT1 plays an important role in the development of lung cancer, Akt/protein kinase B signaling is very important for cancer cell survival and growth when cells are exposed to various apoptotic stimuli. Akt is constitutively activated in NSCLC cells and is a potential target for enhancing the cytotoxicity of chemotherapeutic agents in treatment of NSCLC [51]. Studies have shown that the re-expression of AKT1 can partially rescue the inhibitory effects of miR-185 on the capacity of NSCLC cell proliferation and motility [52]. The over-expression of AKT1 can rescue the effects of miR-548l in NSCLC cells, and the miR-548l expression was inversely correlated with AKT1 expression in NSCLC tissues, indicating that AKT1 was involved in miR-548l-induced suppression of NSCLC cell migration and invasion [53].

In this study, the targets related to taxanes and lung cancer were collected and screened by network pharmacology, and the intersection target of taxanes and lung cancer were analyzed by PPI network analysis, GO biological function enrichment analysis, and KEGG pathway enrichment analysis. To predict the core compounds, targets, pathways, and functions of taxanes in the treatment of lung cancer, eight taxanes-active compounds were screened, corresponding to 444 targets, 738 targets of lung cancer diseases, and 131 intersection target between taxanes and lung cancer. And the top 20 KEGG pathways were obtained. Finally, the top three hub targets with degree value were verified by molecular docking. The results of molecular docking showed that the binding energies of eight taxanes with TP53, EGFR, and AKT1 targets were less than −8.8 kcal/mol. The classical root mean square deviation (RMSD) value was used to assess the ability of each docking tool to predict poses similar to that of the crystallographic structure. RMSD equal to or lower than 2 Å is considered good, 2 Å < RMSD < 3 Å is acceptable and RMSD > 3 Å is bad. However, RMSD < 2 Å cut-off value is widely regarded as the most effective threshold value for validating correctly posed molecules [54]. In this paper, the RMSD values between eight compounds and three targets were all less than 2 Å, indicating that the docking methods and parameters were reasonably designed and that the docking results were highly reliable.

## 5. Conclusions

In the present study, the key active compounds, targets, and pathways of eight taxanes in the treatment of lung cancer were preliminarily predicted by network pharmacology and molecular docking. Eight taxanes mainly act on TP53, EGFR, and AKT1 targets through pathways in cancer, proteoglycans in cancer pathway, and microRNAs in cancer pathway, and plays a role in treating lung cancer in biological functions such as protein binding, enzyme binding, and identical protein binding. Although this study embodies the characteristics of multi-component, multi-target, and multi-pathway, and can provide new ideas for taxanes to treat lung cancer, there are some limitations in network pharmacology. The results need to be further confirmed by related experiments. This paper provides a basic theoretical basis for further study on the key mechanism of taxanes in the treatment of lung cancer in the future.

## Figures and Tables

**Table 1:** Topology analysis of the intersection targets protein–protein interaction.

No.	Gene	Protein Target	Betweenness Centrality	Closeness Centrality	Clustering Coefficient	Degree
1	TP53	Tumor protein p53	0.0651	0.9353	0.4120	121
2	EGFR	Epidermal growth factor receptor	0.0335	0.8904	0.4571	114
3	AKT1	RAC-alpha serine/threonine-protein kinase	0.0271	0.8725	0.4704	111
4	MYC	MYC proto-oncogene, BHLH transcription factor	0.0241	0.8387	0.4996	105
5	ALB	Albumin	0.0337	0.8333	0.4832	104
6	CTNNB1	Catenin beta 1	0.0193	0.8228	0.5218	102
7	VEGFA	Vascular endothelial growth factor A	0.0187	0.8228	0.5205	102
8	STAT3	Signal transducer and activator of transcription 3	0.0139	0.8176	0.5430	101
9	CASP3	Caspase 3	0.0161	0.8075	0.5448	99
10	ERBB2	Erb-B2 receptor tyrosine kinase 2	0.0211	0.8025	0.5092	98
11	IL6	Interleukin 6	0.0150	0.7879	0.5330	95
12	ESR1	Estrogen receptor 1	0.0190	0.7831	0.5427	94
13	PTEN	Phosphatase and tensin homolog	0.0117	0.7784	0.5676	93
14	SRC	SRC proto-oncogene, non-receptor tyrosine kinase	0.0185	0.7784	0.5640	93
15	JUN	Jun proto-oncogene, AP-1 transcription factor subunit	0.0132	0.7738	0.5707	92
16	HIF1A	Hypoxia inducible factor 1 subunit alpha	0.0136	0.7738	0.5791	92
17	EGF	Epidermal growth factor	0.0101	0.7692	0.5819	91
18	CCND1	Cyclin D1	0.0090	0.7647	0.5900	90
19	MAPK3	Mitogen-activated protein kinase 3	0.0102	0.7647	0.5760	90

**Table 2:** Molecular docking results between taxanes and lung cancer targets.

PubChem CID	Compounds	Targets	PDB ID	Uniprot ID	Binding Energy (kcal/mol)
154272	10-DAB III	TP53	6MXY	P04637	−11.3
154272	10-DAB III	EGFR	3POZ	P00533	−11.1
154272	10-DAB III	AKT1	3OS5	P31749	−12.6
65366	Baccatin III	TP53	6MXY	P04637	−12.7
65366	Baccatin III	EGFR	3POZ	P00533	−10.4
65366	Baccatin III	AKT1	3OS5	P31749	−13.7
46783796	7-Xyl-10-DAT	TP53	6MXY	P04637	−9.7
46783796	7-Xyl-10-DAT	EGFR	3POZ	P00533	−8.9
46783796	7-Xyl-10-DAT	AKT1	3OS5	P31749	−9.7
155831	10-DAT	TP53	6MXY	P04637	−11.5
155831	10-DAT	EGFR	3POZ	P00533	−11.0
155831	10-DAT	AKT1	3OS5	P31749	−14.0
148124	Docetaxel	TP53	6MXY	P04637	−10.3
148124	Docetaxel	EGFR	3POZ	P00533	−10.4
148124	Docetaxel	AKT1	3OS5	P31749	−12.9
6436208	CEPH	TP53	6MXY	P04637	−10.1
6436208	CEPH	EGFR	3POZ	P00533	−8.8
6436208	CEPH	AKT1	3OS5	P31749	−11.3
36314	Paclitaxel	TP53	6MXY	P04637	−14.3
36314	Paclitaxel	EGFR	3POZ	P00533	−9.9
36314	Paclitaxel	AKT1	3OS5	P31749	−12.3
184492	7-Epitaxol	TP53	6MXY	P04637	−15.0
184492	7-Epitaxol	EGFR	3POZ	P00533	−9.9
184492	7-Epitaxol	AKT1	3OS5	P31749	−12.3

## Data Availability

The data shown in this study are contained within the article.
